# Cone Beam Computed Tomography Image-Quality Improvement Using “One-Shot” Super-resolution

**DOI:** 10.1007/s10278-024-01346-w

**Published:** 2024-12-04

**Authors:** Takumasa Tsuji, Soichiro Yoshida, Mitsuki Hommyo, Asuka Oyama, Shinobu Kumagai, Kenshiro Shiraishi, Jun’ichi Kotoku

**Affiliations:** 1 Graduate School of Medical Care and Technology, Teikyo University, 2-11-1 Kaga, Itabashi-Ku, Tokyo, 173-8605 Japan; 2 Department of Radiology, The University of Tokyo Hospital, 7-3-1 Hongo, Bunkyo-Ku, Tokyo, 113-8655 Japan; 3 Health Care Division, Health and Counseling Center, Osaka University, 1 Machikaneyamatyo, Toyonaka-Shi, Osaka, 560-0043 Japan; 4 Central of Radiology, Teikyo University Hospital, 2-11-1 Kaga, Itabashi-Ku, Tokyo, 173-8606 Japan; 5 Department of Radiology, Teikyo University School of Medicine, 2-11-1 Kaga, Itabashi-Ku, Tokyo, 173-8605 Japan

**Keywords:** Cone beam CT, Deep learning, Deformable image registration, One-shot learning, Super-resolution

## Abstract

Cone beam computed tomography (CBCT) images are convenient representations for obtaining information about patients’ internal organs, but their lower image quality than those of treatment planning CT images constitutes an important shortcoming. Several proposed CBCT image-quality improvement methods based on deep learning require large amounts of training data. Our newly developed model using a super-resolution method, “one-shot” super-resolution (OSSR) based on the “zero-shot” super-resolution method, requires only small amounts of training data to improve CBCT image quality using only the target CBCT image and the paired treatment planning CT image. For this study, pelvic CBCT images and treatment planning CT images of 30 prostate cancer patients were used. We calculated the root mean squared error (RMSE), peak signal-to-noise ratio (PSNR), and structural similarity (SSIM) to evaluate image-quality improvement and normalized mutual information (NMI) as a quantitative evaluation of positional accuracy. Our proposed method can improve CBCT image quality without requiring large amounts of training data. After applying our proposed method, the resulting RMSE, PSNR, SSIM, and NMI between the CBCT images and the treatment planning CT images were as much as 0.86, 1.05, 1.03, and 1.31 times better than those obtained without using our proposed method. By comparison, CycleGAN exhibited values of 0.91, 1.03, 1.02, and 1.16. The proposed method achieved performance equivalent to that of CycleGAN, which requires images from approximately 30 patients for training. Findings demonstrated improvement of CBCT image quality using only the target CBCT images and the paired treatment planning CT images.

**Supplementary Information:**

The online version contains supplementary material available at 10.1007/s10278-024-01346-w.

## Introduction

Cone beam computed tomography (CBCT) has been used increasingly in recent years because radiation therapy has come to include high-precision irradiation, known as image-guided radiotherapy (IGRT), such as stereotactic radiotherapy, intensity-modulated radiotherapy, and volumetrically modulated arc therapy. In particular, CBCT is a valuable tool for IGRT because it is attached to radiation irradiation devices. Moreover, it can quickly produce the latest information related to the locations of targets and organs inside a patient [1, 2].

Adaptive radiation therapy (ART), a more precise form of radiation therapy, has been introduced recently. Yan et al. proposed the ART method in the late 1990s [3]. The treatment plan is reexamined sequentially during the treatment period, considering tumor weight loss and shrinkage caused by treatment. A combination of radiation therapy devices and superconducting low magnetic field generators has been developed and introduced into clinical practice [4–6]. Magnetic resonance imaging is used before treatment to evaluate the dose distribution in the body immediately before treatment and to optimize the treatment plan. This imaging ensures that a maximum dose is given to tumor tissues and that a minimum dose is given to normal tissues. However, the introduction of magnetic resonance image-guided radiation equipment presents some difficulties. Introducing new equipment is expensive. Moreover, some patients cannot enter the gantry because of claustrophobia or metal in their bodies [7].

For treatment replanning in ART, CBCT is suitable because it is applied immediately after the start of irradiation and because it can readily acquire positional information of targets and organs inside the patient. Nevertheless, an important shortcoming of CBCT is that the image quality is inadequate compared to treatment planning CT images. Because of its imaging principle, CBCT images have low contrast attributable to artifacts and scattered rays [8–10]. In practice, calculating the dose distribution necessary for treatment planning directly from low-quality images such as CBCT images is extremely difficult.

Some methods have been proposed to use low-quality CBCT images for ART by first superimposing treatment planning CT images on CBCT images using a non-rigid transformation and by then replanning the treatment [11–13]. These methods can create high-quality treatment planning CT images using the latest internal information of patients. It can subsequently facilitate treatment replanning. However, guaranteeing high positional accuracy in areas with large positional deviations between images, such as cavities and soft tissues in the intestinal tract, is difficult.

Many methods have been developed to improve CBCT image quality for use in ART. For instance, some methods improve image quality by reducing artifacts caused by reconstruction errors. The methods achieve that improvement by correcting the scattered ray components in CBCT images [14–16]. Unfortunately, these methods require special filters to improve image quality and require preprocessing before image reconstruction.

Several deep learning–based CBCT image-quality improvement methods have been proposed recently [17–23]. For example, Kida et al. proposed a method based on U-net [24]. This method uses 15 pairs of CBCT images and treatment planning CT images of 20 prostate cancer patients for training [17]. Other methods that apply CycleGAN [25, 26] have been proposed, such as a method described by Ozaki et al. using megavoltage CT images and kilovoltage CT images of head and neck cancer patients [18] and the method presented by Liu et al. using pairs of CBCT images and treatment planning CT images of 30 pancreatic cancer patients for training [19]. In addition, using dictionary learning, Oyama et al. improved the CBCT image quality using only a small amount of data [27].

Furthermore, a method reported by Kida et al. uses CBCT images and treatment planning CT images of 16 prostate cancer patients for training [20]. A method described by Kurz et al. uses CBCT images and treatment planning CT images of 25 of 33 prostate cancer patients for training [21]. Consequently, the deep learning–based method applies to the processing of the reconstructed image and achieves a certain level of quality improvement using no special filter. Nevertheless, all these methods require the use of large amounts of training data. These methods are generally not easy to use because obtaining large amounts of medical data is difficult.

Super-resolution technology has recently developed a method for estimating and generating a high-resolution image from a low-resolution image. Particularly, method-based deep learning has power and great effectiveness. The first super-resolution model-based deep learning was reported in 2015: the super-resolution convolutional neural network (SRCNN) [28]. This learning method was followed by a fast super-resolution convolutional neural network (FRCNN) and a smaller and faster convolutional neural network (CNN) achieved by adding an up-sampling layer and by increasing resolution through deconvolution [29]. Moreover, many super-resolution models based on deep learning have been reported. For example, one method deepens the CNN to 20 layers by incorporating the ResNet [30] method [31] and residual channel attention networks (RCAN) that achieve high performance using an attention network. That method can thereby identify the features to examine at each layer specifically and can add them as attention information [32].

As super-resolution technique-based deep learning continues to develop, some reports have described methods that require no large amounts of training data in advance and which use only a single test image to achieve super-resolution [33–35]. These methods present the benefits of being versatile and of being applicable to any image. If these super-resolution techniques, which require no large amount of pre-training data, were applicable to CBCT images, then these methods would be adequate to resolve difficulties posed by existing methods requiring large amounts of training data.

For this study, we developed a new one-shot CBCT image-quality improvement model based on “zero-shot” super-resolution (ZSSR) [33]. This method requires only small amounts of pre-training data and uses only the treatment planning CT images paired with the target CBCT images.

## Materials and Methods

### Data Acquisition

After anonymization, whole-pelvic-region images taken between May 27, 2015, and January 16, 2017, at Teikyo University Hospital were used for this study. The CBCT images (On-Board Imager; Varian Medical Systems, Inc., Tokyo, Japan) and treatment planning CT images (Aquilion LB; Canon Medical Systems Corp., Tochigi, Japan) were from 30 prostate cancer patients. These CBCT images were acquired on the first day of radiotherapy. This study was approved by the institutional ethics review board (Teikyo University Review Board 17–108-6). The need for written informed consent from patients was waived because the patient data had been anonymized.

Table 1 presents the imaging parameters of CBCT images and treatment planning CT images used for this study. Each value represents the minimum and maximum value: the only value indicating that the minimum and maximum values are equal. Both CBCT and treatment planning CT images have image sizes of 384 × 384 pixel.

**Table 1:** Imaging parameters of CBCT images and treatment planning CBCT images

	CBCT	Treatment planning CT
Slice thickness (mm)	2.5	2.0
Pixel width (mm)	1.172	0.877–1.074
X-ray tube voltage (kVp)	125	120
X-ray exposure (mAs)	679–686	250–600

### Gradation Processing

Acquired CBCT images and treatment planning CT images are digital imaging and communications in medicine (DICOM) images with 16 bits per pixel. The pixel values of the DICOM image (*D*) were converted to CT values (*C*). The CT values are defined on a scale of 1000 for calcium, 0 for water, and − 1000 for air. The CT values were calculated using the rescaled intercept (${R}_{\text{inter}}$) and rescaled slope (${R}_{\text{slope}}$) obtained from the DICOM image as

1 $$C={R}_{\text{slope}} D+{R}_{\text{inter}}.$$

Next, the calculated CT values were converted to pixel values (*P*) as

2 $$P={255}^{2}\times \frac{\left(C-m\right)}{{W}_{w}}.$$

If the pixel value (*P*) is greater than 255^2^, then it is set as 255^2^. If it is less than 0, then it is set as 0, resulting in a 16-bit image from 0 to 255^2^. Here, $m$ stands for the minimum value of the window, which is calculated using the window center (${W}_{c}$) and window width (${W}_{w}$) as

3 $$m={W}_{c}-\frac{{W}_{w}}{2}.$$

We followed the process described above for the CBCT images. Additionally, we applied the same process for the treatment planning CT images using the value obtained from the CBCT images of the same patient.

### Image Registration and Pairing of CBCT Images and Treatment Planning CT Images

The acquired CBCT images and treatment planning CT images differed in slice thickness and in the number of slices per patient. The CBCT images had a 2.5-mm slice thickness. The number of slices per patient was 64. By contrast, treatment planning CT images had a 2.0-mm slice thickness. The number of slices per patient varied from 129 to 178 (median, 153). Consequently, we created 64 pairs of CBCT images and treatment planning CT images per patient by referring to visual inspection and the slice levels retrieved from DICOM, according to the number of CBCT images. Three licensed radiological technologists selected the treatment planning CT slice corresponding to the organs in the CBCT images. The final pairs of CBCT and treatment planning CT images were determined by majority voting by the three annotators. In cases where each of the three selected a different slice, the median slice position was chosen from the three annotations.

**Fig. 1:**
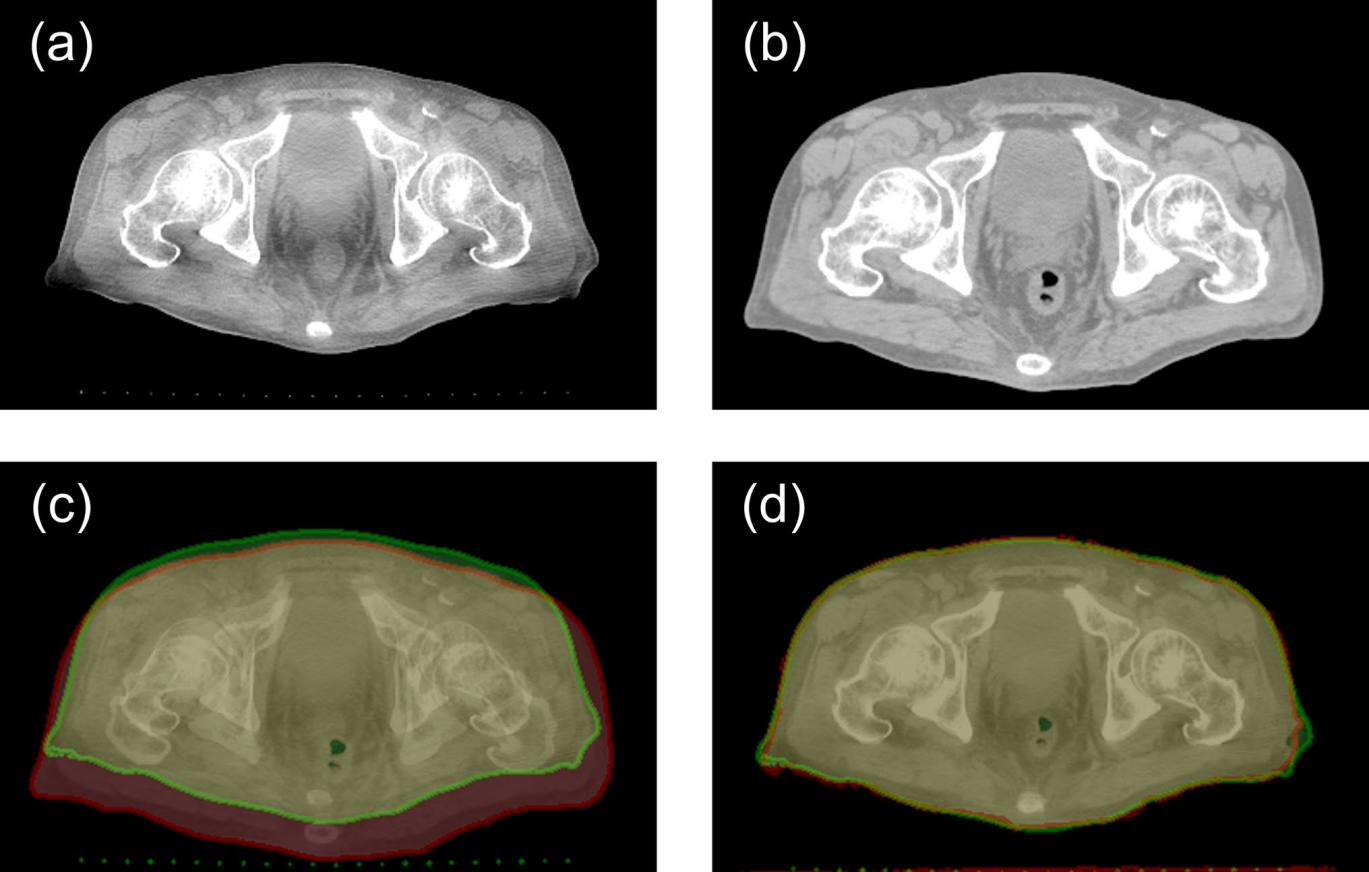
Results of superimposition of treatment planning CT images on CBCT images by B-spline deformable registration: CBCT image as a low-resolution image (**a**), treatment planning CT image as a high-resolution image (**b**), alpha blending image of CBCT image (green) and treatment planning CT image (red) before registration (**c**), and alpha blending image after registration (**d**)

For each pair, we regarded the CBCT images as low-resolution images and regarded the treatment planning CT images as high-resolution images. The treatment planning CT images were superimposed on the corresponding CBCT images by B-spline deformable registration using SimpleITK open-source software [36–38]. The obtained results are presented in Fig. 1.

### Proposed Method Based on “Zero-Shot” Super-resolution

#### Overview of “Zero-Shot” Super-resolution

The “zero-shot” super-resolution (ZSSR) method, proposed by Shocher et al. in 2018, leverages deep learning. It does not rely on prior learning [33]. The method, commonly called internal learning, uses only a single test image. The method takes a high-resolution image extracted from the test image and downscales it to create a low-resolution image. The low-resolution image is input to the small image-specific CNN (SimpleNet), which is trained to minimize the mean absolute error between the output and high-resolution images. The super-resolution image can then be estimated by inputting a test image to this trained CNN. Therefore, ZSSR resolves some difficulties posed by earlier deep learning-based super-resolution methods. The performance of those earlier methods is degraded considerably when the degradation of the training data and that of the test data differ. In other words, ZSSR is applicable to different settings for each image. It can also perform super-resolution even for images for which the shooting process is unknown or a non-idea, such as actual old photographs, noisy photographs, and biological data.

#### Overview of the One-Shot Super-resolution Method

**Fig. 2:**
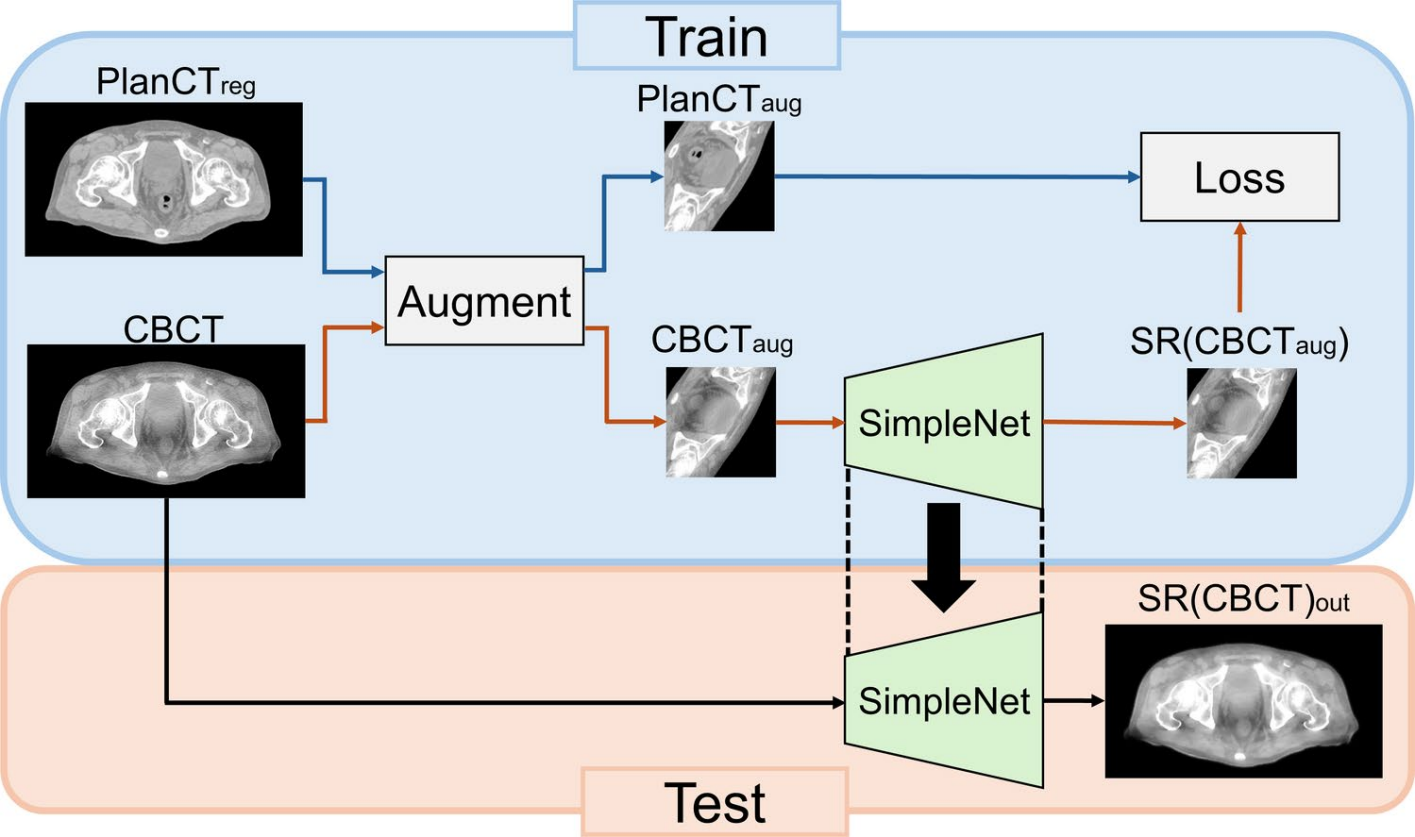
Schematic drawing of our proposed OSSR algorithm. Loss is the mean squared error (MSE). The following notation is used: ${\text{PlanCT}}_{\text{reg}}$, treatment planning CT image after B-spline deformable registration; ${\text{PlanCT}}_{\text{aug}}$, data-enhanced treatment planning CT image; ${\text{CBCT}}_{\text{aug}}$, data-enhanced CBCT image; $\text{LR}({\text{CBCT}}_{\text{aug}})$, lower-quality image of ${\text{CBCT}}_{\text{aug}}$; $\text{SR}({\text{CBCT}}_{\text{aug}})$, SimpleNet output image of $\text{LR}({\text{CBCT}}_{\text{aug}})$; ${\text{SR}(\text{CBCT})}_{\text{out}}$, SimpleNet output image-quality improvement image of the target CBCT image

The ZSSR presented in the preceding section constructed a pseudo-low-resolution–high-resolution image pair from the internal information of a single test image and constructed a super-resolution model specific to that test image. We propose a one-shot super-resolution (OSSR) that can convert a CBCT image to a treatment planning CT image by replacing this pair of low-resolution and high-resolution images with a CBCT image and treatment planning CT image, using only these images. A schematic drawing of the proposed method is depicted in Fig. 2.

**Fig. 3:**
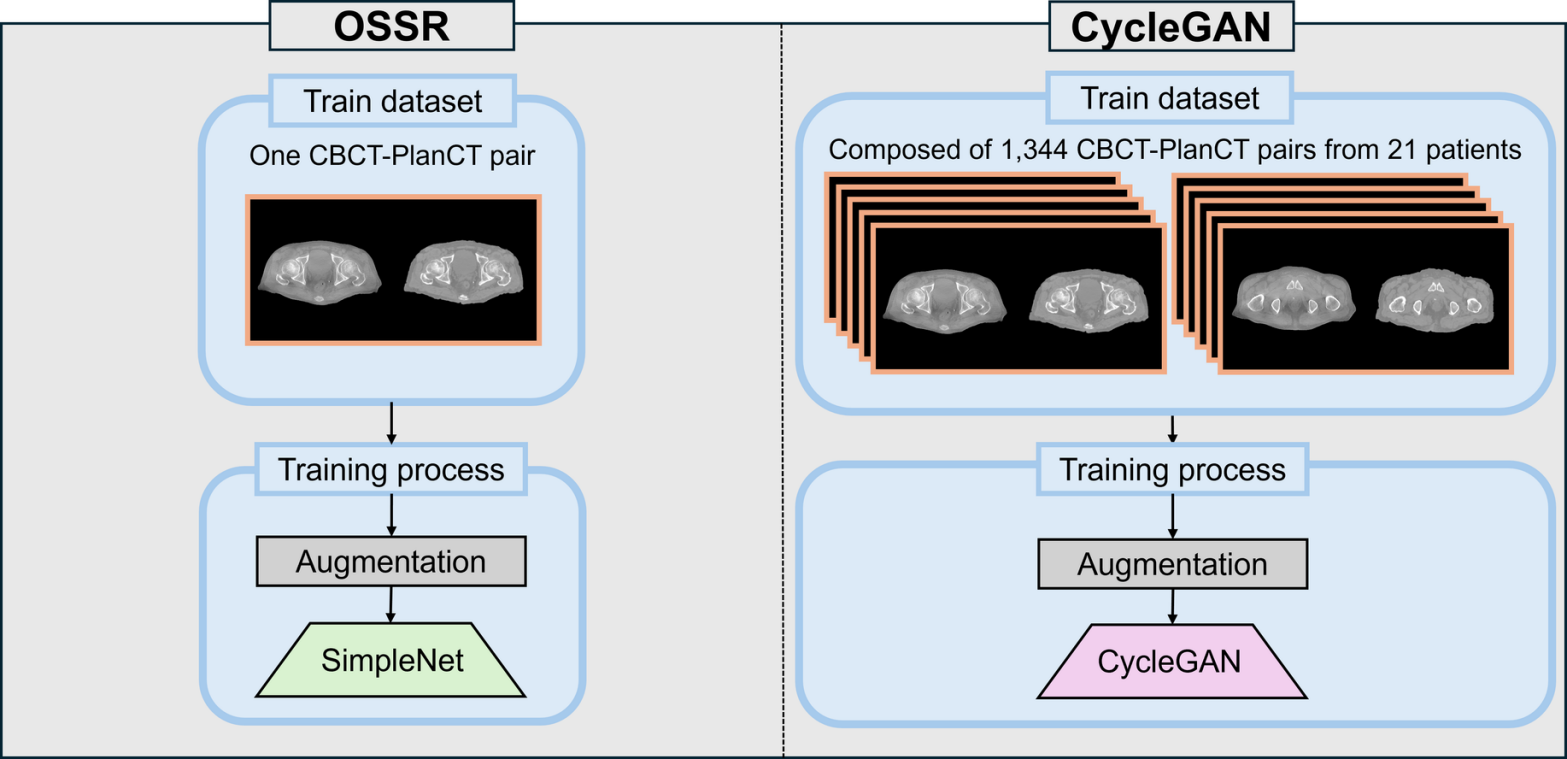
Comparison of the training dataset used for the training of OSSR and the conventional learning method CycleGAN. OSSR trains the model using only a single CBCT–PlanCT pair, whereas CycleGAN trains the model with 1344 CBCT–PlanCT pairs from 21 patients

An outline of the data-splitting process for inputting CBCT-treatment planning CT pairs into OSSR is depicted in Fig. 3. The only training dataset used for this method was a pair of CBCT images and treatment planning CT images. Therefore, we first performed data expansion of the target CBCT image and treatment planning CT image pairs (CBCT–PlanCT pairs) to train on more CBCT–PlanCT pairs. The data expansion was a random combination of rotation (0°, 90°, 180°, 270°), left–right flip, and crop (128 × 128 image size, centered on a random location in the image) to increase the number of CBCT–PlanCT pairs. The training dataset comprises many target CBCT–PlanCT pairs obtained through data expansion. Second, we train the network (SimpleNet) using this training dataset to reduce the mean squared error (MSE) between the CBCT–PlanCT pairs. The trained SimpleNet estimated the image-quality improvement image of the target CBCT image. The proposed model removes the downscaling process included in the original ZSSR [33]. Details of this method are presented in the following sections.

### SimpleNet

**Fig. 4:**
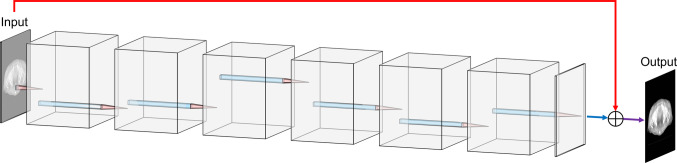
SimpleNet structure. This figure was created using NN-SVG [39]

SimpleNet has eight convolutional layers, with an activation function (rectified linear unit, ReLU) applied to each layer. In addition, SimpleNet has a skip connection [30]. Therefore, the original image structure is not lost. Because the SimpleNet output size is the same as the input image size, it is applicable to images of different sizes. The SimpleNet structure is portrayed by LeNail [39] as depicted in Fig. 4. Details of the SimpleNet structure are presented in Table 2.

**Table 2:** SimpleNet structure output size: *ch*, input image number of channels; *h*, input image height; *w*, input image width

Layer	Kernel size	Stride	Padding	Output size (*ch*, *h*, *w*)
Input	$-$	$-$	$-$	(1, *h*, *w*)
ReLU + Conv1	(3, 3)	(1, 1)	(1, 1)	(128, *h*, *w*)
ReLU + Conv2	(3, 3)	(1, 1)	(1, 1)	(128, *h*, *w*)
ReLU + Conv3	(3, 3)	(1, 1)	(1, 1)	(128, *h*, *w*)
ReLU + Conv4	(3, 3)	(1, 1)	(1, 1)	(128, *h*, *w*)
ReLU + Conv5	(3, 3)	(1, 1)	(1, 1)	(128, *h*, *w*)
ReLU + Conv6	(3, 3)	(1, 1)	(1, 1)	(128, *h*, *w*)
ReLU + Conv7	(3, 3)	(1, 1)	(1, 1)	(128, *h*, *w*)
ReLU + Conv8	(3, 3)	(1, 1)	(1, 1)	(1, *h*, *w*)
Residual	-	-	-	(1, *h*, *w*)
Output	-	-	-	(1, *h*, *w*)

#### SimpleNet Training

We trained SimpleNet using our created training dataset. The optimization algorithm was Adam. The initial value of the learning rate was set as 1.0 × 10^−3^. Periodically, a linear fit of the reconstruction error is performed. If the standard deviation is greater than the slope of the linear fit, then the learning rate is divided by 10 [33]. Learning was terminated when the learning rate reached 1.0 × 10^−6^.

#### Creation of Image-Quality Improvement Images of Target CBCT Images

We used the trained SimpleNet to create a quality improvement image of the target CBCT image. First, we generated eight images by combining rotation (0°, 90°, 180°, 270°) and left–right flipping of the target CBCT image. Next, the eight generated images were input to the trained SimpleNet. Eight images were output. The eight output images from SimpleNet were returned to the original image direction. The median of the eight returned images was taken as the final output image from SimpleNet. Here, the pixel values of the images input to SimpleNet were normalized to 0–1. For this OSSR method, one SimpleNet is trained on one CBCT image and one PlanCT image. Therefore, training was performed for the number of slices for one patient.

We built the proposed network on a Deep Learning Box II running on a computer (Xeon GPUs; Intel Corp. and RTX A6000 48 GB GPU; NVIDIA Corp.) with a PyTorch (ver. 1.12.1) deep learning framework. As a comparison of the OSSR of the proposed method, we conducted a comparison using total variation denoising (TVD) [40], a filtering process, and CycleGAN [25], which is often used as a method for improving CBCT image quality. An overview of CycleGAN and the learning conditions is presented in the following section.

### Comparison of the Proposed Method with CycleGAN

We compared the accuracy of the proposed OSSR method by evaluating CycleGAN [25], which is often used for image-quality improvement studies of CBCT images on a similar dataset. CycleGAN is a network based on adversarial networks that transform paired images belonging to different domains in each other’s domain. In medical imaging, CycleGAN can improve image quality for unpaired datasets while preserving anatomical information. However, large amounts of training data must be used to train the model.

In applying CycleGAN in medical imaging, Wolterink et al. devised a method to convert MRI images to CT images to introduce MRI images without risk of radiation exposure in radiotherapy planning [41]. Ozaki et al. based their proposed method for converting MVCT images to kVCT images on CycleGAN [18]. For the present study, we evaluated the image-quality improvement of converting CBCT images to the domain of treatment planning CT images using CycleGAN, which replaced the domain transformation of the original CycleGAN proposed by Zhu et al. [25] to CBCT and treatment planning CT images by Liang et al. [26]. Details of the CycleGAN network structure and loss function setting used for this analysis are provided as Supplementary Information (Supplementary File).

As a preprocessing step to input images to CycleGAN, windowing was performed during the gradation processing and preprocessing of the proposed method. The resulting images were scaled from − 1 to 1 and input to the CycleGAN. The CBCT and treatment planning CT images were subjected to the same vertical and horizontal flipping conditions for data augmentation.

We applied the commonly used machine learning model evaluation method of group tenfold cross-validation to evaluate CycleGAN training. This method involved dividing 30 patients into 10 folds, each consisting of 3 patients; each patient had 64 CBCT and treatment planning CT images. The remaining 7 and 2 folds were used respectively as a training dataset (comprising 21 patients with a total of 1344 pairs) and a validation dataset (comprising 6 patients with 384 pairs). The slitting, training, and evaluation process was repeated ten times, ensuring that each group served once as the test dataset. Additionally, we ensured that no patients overlapped across the training, validation, and test datasets.

For the training of CycleGAN, we used the Adam optimizer as the optimization algorithm to perform scratch training across all networks for 100 epochs. The batch size was set as 1. To prevent model overfitting, early stopping was implemented when the MSE between the treatment planning CT in the validation data and the synthetic treatment planning CT images generated from CBCT images reached its minimum.

### Quantitative Evaluation

As quantitative evaluation of the positioning accuracy by application of the proposed method, we calculated the normalized mutual information (NMI) [42] of the treatment planning CT image after B-spline deformable registration (PlanCT_reg_) and of the CBCT image after application of the proposed method (SR(CBCT)_out_) and NMI between PlanCT_reg_ and the CBCT image before application of the proposed method. For similarity assessment between images obtained from different modalities, NMI is used widely. A larger value represents the higher similarity between two images.

In addition, as a quantitative evaluation of the image-quality improvement by application of the proposed method, we calculated four metrics between the CBCT image before the application of the proposed method and PlanCT_reg_ and between the SR(CBCT)_out_ and PlanCT_reg_. These three metrics are the root mean squared error (RMSE), peak signal-to-noise ratio (PSNR), and structural similarity (SSIM). The RMSE is the standard deviation of the residuals between the two images. PSNR represents the reduction of noise between two images. SSIM is an index of differences perceived by humans more accurately than the PSNR because it calculates overall similarity from similarity of three types: luminance-based similarity, contrast-based similarity, and structure-based similarity [43]. Higher similarity between two images is associated with a lower RMSE value and higher PSNR and SSIM values.

We performed statistical tests to calculate the significance of the analysis results. Because the data are paired, we conducted repeated measures ANOVA for more than four groups: PlanCT/CBCT, TVD, CycleGAN, and OSSR. Subsequently, paired *t*-tests were conducted for each pair of groups. For these tests, a *p* value < 0.001 was inferred as significant.

## Results

**Fig. 5:**
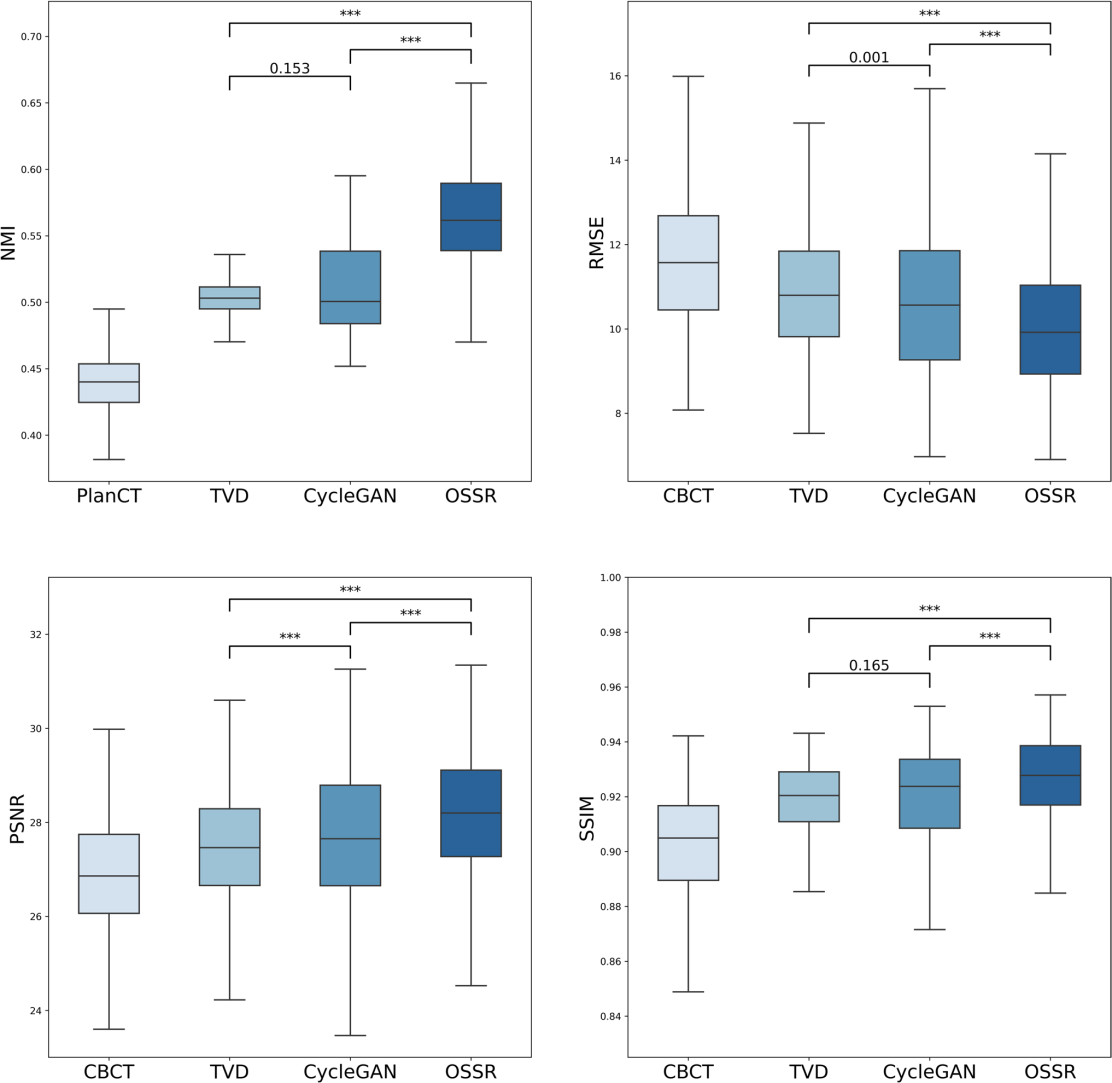
Quantitative evaluation by NMI (upper left), RMSE (upper right), PSNR (lower left), and SSIM (lower right) of pelvic CT images of 30 patients (box plot). We created this figure using the average value per patient. Using repeated measures ANOVA, these four groups were found to have significant differences (*p* value < 0.001). Additionally, paired *t*-tests were applied for each pair of the four groups. The results revealed significant differences (*p* value < 0.001) for all evaluation metrics in all combinations, except for the combination of TVD and CycleGAN. ***p value < 0.001

We calculated NMI as a quantitative evaluation of positional accuracy by application of the proposed method. We also calculated RMSE, PSNR, and SSIM for the quantitative evaluation of image-quality improvement by applying the proposed method. Table 3 presents the mean and standard deviation of these results for all data. Additionally, these results are presented as box plots in Fig. 5. Each patient had 64 CBCT–PlanCT pairs. Subsequently, after performing each quantitative evaluation of all 64 pairs for each patient, we calculated each value. Comparison results of quantitative evaluation by NMI, RMSE, PSNR, and SSIM for each patient are presented in Supplementary Figs. 1–4 (Supplementary File).

**Table 3:** Quantitative evaluation of image-quality improvement by NMI, RMSE, PSNR, and SSIM

Model	NMI↑	RMSE↓	PSNR↑	SSIM↑
CBCT	0.439 ± 0.019 (0.394–0.471)	11.70 ± 1.09 (10.02–13.72)	26.85 ± 0.81 (25.40–28.12)	0.902 ± 0.018 (0.853–0.928)
TVD	0.502 ± 0.012 (0.472–0.523)	10.95 ± 0.99 (9.50–12.85)	27.42 ± 0.78 (25.97–28.59)	0.919 ± 0.009 (0.900–0.933)
CycleGAN	0.510 ± 0.031 (0.461–0.575)	10.64 ± 1.28 (7.98–12.72)	27.72 ± 1.08 (26.06–30.12)	0.921 ± 0.014 (0.890–0.942)
OSSR	0.575 ± 0.030 (0.526–0.637)	10.08 ± 1.01 (8.51–11.82)	28.15 ± 0.86 (26.69–29.56)	0.926 ± 0.012 (0.897–0.944)

Values in the table represent the mean and standard deviation (min–max): CBCT, low-resolution images (NMI is calculated with respect to the treatment planning CT images after registration using B-spline deformable registration); TVD, CBCT images with total variation denoising; CycleGAN, synthetic PlanCT images converted from CBCT images by generating CycleGAN; OSSR, image-quality improvement images generated from CBCT images using the proposed method based on OSSR

The value of NMI between the treatment planning CT images after registration using B-spline deformable registration and the CBCT images before application of the proposed method was 0.439 ± 0.019, on average, for each patient. Similarly, the NMI values between TVD and CBCT images and between CycleGAN and CBCT images were, respectively, 0.502 ± 0.012 and 0.510 ± 0.031, whereas the NMI between SR(CBCT)_out_ images before the application of the proposed method were 0.575 ± 0.030, on average, for each patient. These NMI results were found to have significant differences (*p* value < 0.001), except for the combination of TVD and CycleGAN.

The average RMSE of CBCT images before application of the proposed method and treatment planning CT images was 11.70 ± 1.09 for each patient. In contrast, each patient’s average RMSE of between treatment planning CT images and the CBCT images generated by TVD, CycleGAN, and SR(CBCT)_out_ output by the proposed method were, respectively, 10.95 ± 0.99, 10.64 ± 1.28, and 10.08 ± 1.01. These RMSE values were found to have significant differences (*p* value < 0.001) in all combinations, except for TVD and CycleGAN. These results indicate that the application of TVD, CycleGAN, and the proposed method improved RMSE relative to the CBCT images, with respective reductions to averages of 0.94 times, 0.91 times, and 0.86 times across all patients.

The average PSNR of CBCT images before application of the proposed method and treatment planning CT images was 26.85 ± 0.81 for each patient. In contrast, the average PSNRs between treatment planning CT images and CBCT images by TVD, CycleGAN, and SR(CBCT)_out_ generated using the proposed method were, respectively, 27.42 ± 0.78, 27.52 ± 1.08, and 28.15 ± 0.86. Significant differences in PSNR (*p* value < 0.001) were observed across all combinations. These findings suggest that the application of TVD, CycleGAN, and the proposed method enhanced PSNR compared to the CBCT images, with an average improvement of 1.02 times, 1.03 times, and 1.05 times, respectively, across all patients.

The average SSIM of CBCT images before application of the proposed method and treatment planning CT images was 0.902 ± 0.018 for each patient. In contrast, the average SSIMs between treatment planning CT images and CBCT images by TVD, CycleGAN, and SR(CBCT)_out_ generated using the proposed method were, respectively, 0.919 ± 0.009, 0.921 ± 0.014, and 0.926 ± 0.012. All combinations, except for TVD and CycleGAN, demonstrated significant differences in SSIM (*p* value < 0.001). The results demonstrated that the application of TVD, CycleGAN, and the proposed method led to improved SSIM relative to the CBCT images, with average increases of 1.02 times, 1.02 times, and 1.03 times, respectively, for all patients. These results indicate that the application of the proposed method improved the values of RMSE, PSNR, and SSIM to yield performance that is comparable to that provided by CycleGAN.

**Fig. 6:**
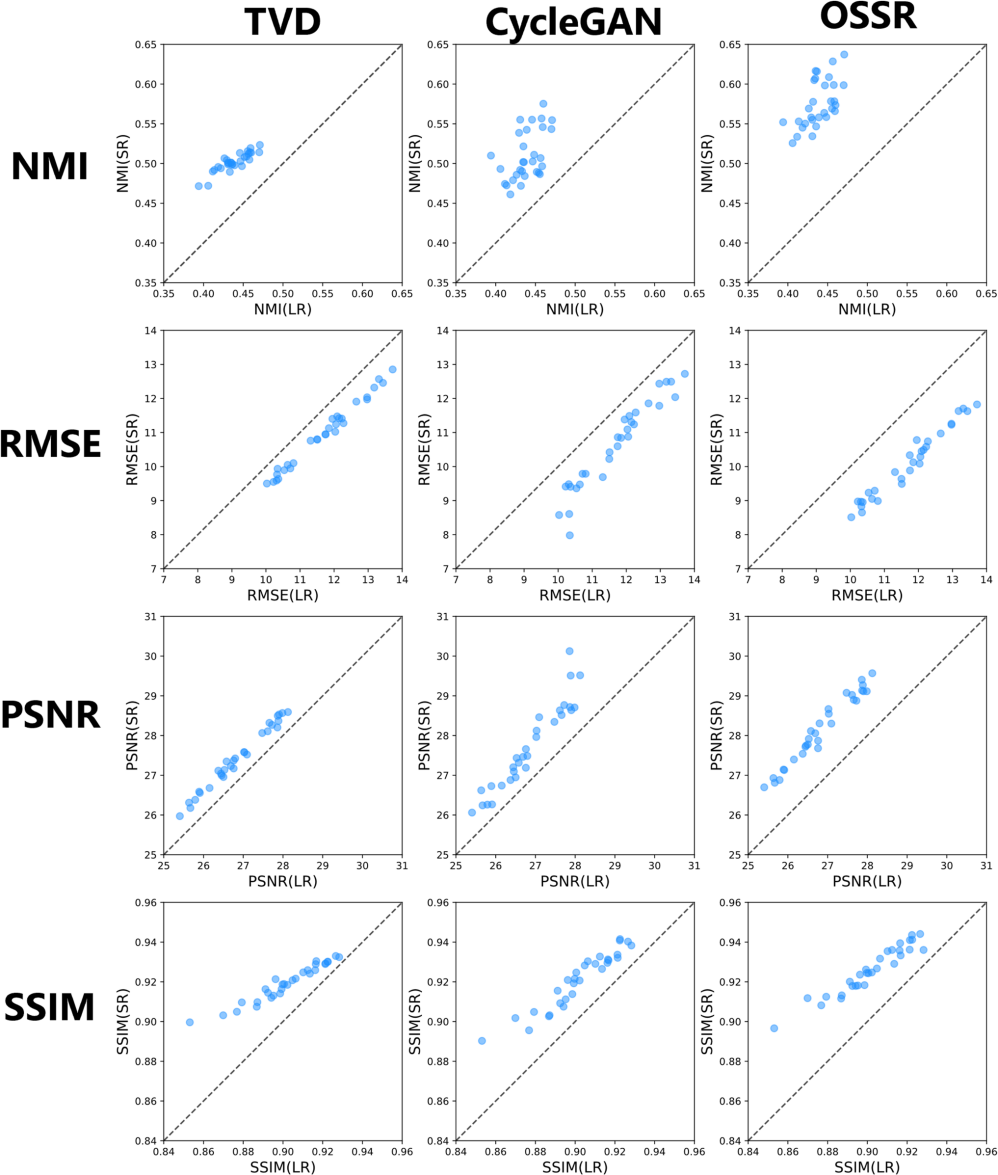
Quantitative evaluation by NMI (top), RMSE (upper middle), PSNR (lower middle), and SSIM (bottom) of pelvic CT images of 30 patients (scatter plot). The left, center, and right columns, respectively, show scatter plots of TVD, CycleGAN, and the proposed method (OSSR). Each quantitative evaluation value for all 1920 images is shown. If the image quality did not differ before and after the application of the proposed method, then the marker is shown on the dashed line. The horizontal axis shows the value of quantitative evaluation between the CBCT image before the application of the proposed method and the treatment planning CT image. The vertical axis shows the value of quantitative evaluation between the SR(CBCT)_out_ and the treatment planning CT image

Regarding RMSE and PSNR improvement, TVD showed improvement in 1918 of 1920 images (99.90%), and CycleGAN showed improvement in 1906 of 1920 images (99.27%), whereas the proposed method improved 1916 of 1920 images (99.79%). Similarly, for SSIM, whereas TVD achieved improvement in 1877 of 1920 images (97.76%), CycleGAN achieved improvement across all images (100%), and the proposed method achieved improvement in 1892 of 1920 images (98.54%). Regarding the evaluation per image, the results of the comparison of quantitative evaluations by RMSE, PSNR, and SSIM for all 1920 images (for 30 patients) are presented in Fig. 6.

**Fig. 7:**
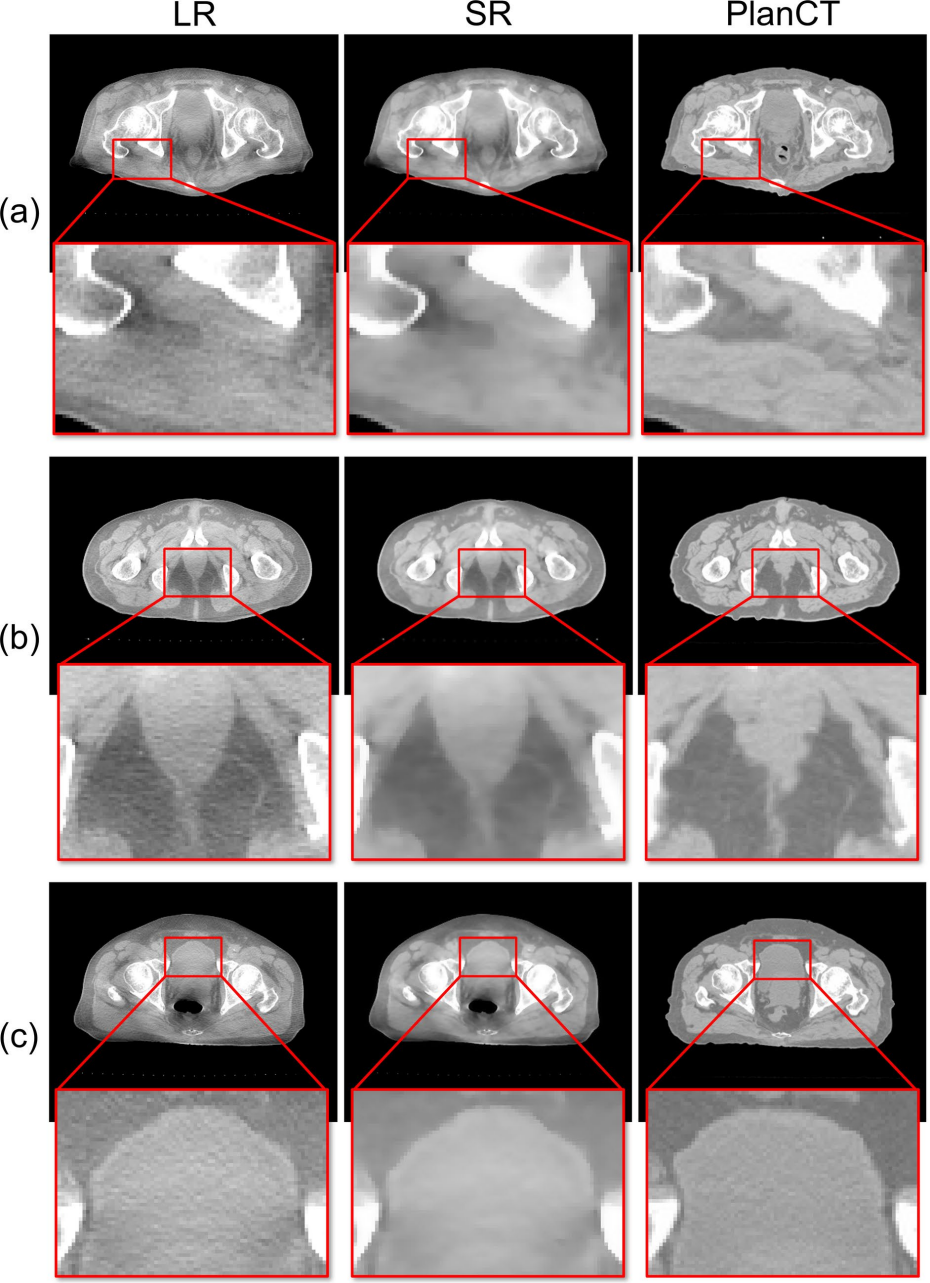
Exemplary images of application to pelvis CT images. CBCT images before processing (LR column). Processed image-quality improvement images (SR column). Treatment planning images with the same slice as the CBCT image (PlanCT column). **a**, **b**, and **c**, respectively, show images of different patients, depicting enlarged images of muscle and fat (**a**), rectum (**b**), and bladder (**c**). The CT number display window ranges from [− 840, 600] HU

**Fig. 8:**
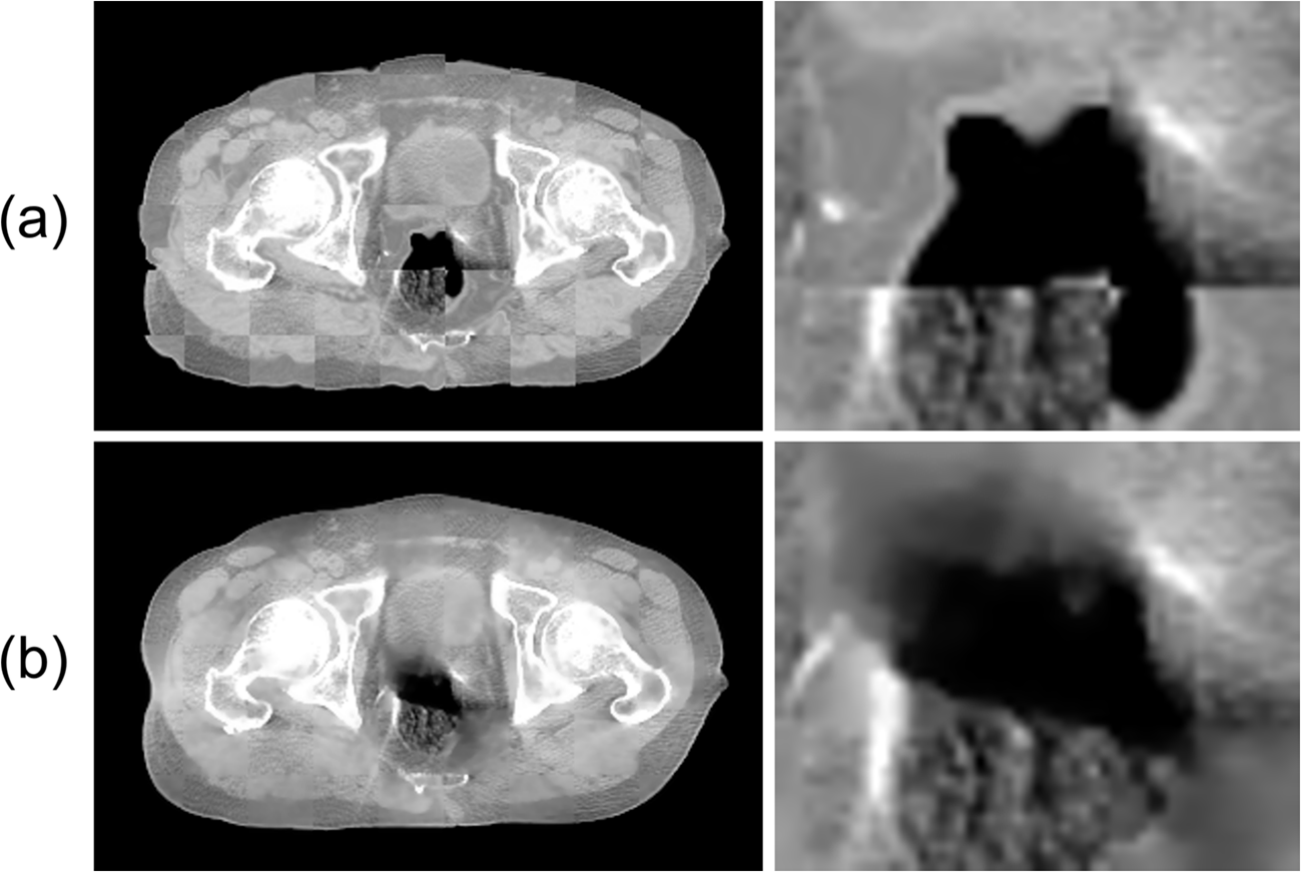
Checkerboard images. Checkerboard image of CBCT image and treatment planning CT image after B-spline deformable registration (**a**). Checkerboard image of CBCT image and SR(CBCT)_out_. Each right image is an enlarged rectal area (**b**) image. The CT number display window ranges from [− 840, 600] HU

Results obtained from the application of the proposed method are presented in Fig. 7. The checkerboard image is presented in Fig. 8. When the treatment planning CT image was superimposed on the CBCT image using B-spline deformable registration, the positioning accuracy was low, especially for the internal cavity in the rectal area. By contrast, the proposed method achieved high positioning accuracy, especially for internal cavities such as the rectum. Moreover, the proposed method suppressed artifacts and enhanced contours.

Actually, NMI was found to have an average improvement of 1.14 times with TVD, 1.16 times with CycleGAN, and 1.31 times with the proposed method across all patients. These results demonstrate that the proposed method achieves higher visual and quantitative positional accuracy than conventional methods, which use only B-spline deformable registration [11–13].

## Discussion

A shortcoming of CBCT images is that, because of the method’s imaging principle, scattered rays degrade the image quality. Deep learning–based CBCT image-quality improvement methods have been proposed to resolve this shortcoming, but such methods require large amounts of pre-training data. As described herein, we newly constructed a one-shot image-quality improvement model based on ZSSR and presented a method for improving the CBCT image quality using only treatment planning CT images paired with target CBCT images. When applying the proposed method, the results of RMSE, PSNR, and SSIM in the CBCT images were as much as 0.86 times, 1.05 times, and 1.03 times better, on average, than those obtained from the treatment planning CT images obtained without using the proposed method. In addition, the NMI result obtained for CBCT images when applying the proposed method was as much as 1.31 times better than that obtained from the treatment planning CT images obtained without using the proposed method. In addition, high positional accuracy was achieved from visual evaluation. A comparison to the proposed method shows that the RMSE, PSNR, SSIM, and NMI results for CycleGAN, which requires large amounts of training data, were, respectively, only 0.91, 1.03, 1.02, and 1.16 times improved. These results demonstrate that the proposed method achieves an equivalent level of accuracy to that of CycleGAN, which performs well in terms of image-quality improvement and high positioning accuracy of CBCT images. However, the proposed method does so using only a pair of treatment planning CT images and target CBCT images. Additionally, the proposed method outperformed the filtering method TVD.

The visual and quantitative evaluation results achieved by the application of the proposed method demonstrated a clear improvement in image quality. However, out of the 1920 images, 43 (2.24%) showed no improvement in image quality on RMSE and PSNR, and 28 (1.46%) showed none on SSIM.

As quantitative evaluation by NMI and Fig. 8 show, the proposed method achieved higher guaranteed positional accuracy than when using only B-spline deformable registration. These results demonstrate that the use of only a pair of CBCT images and treatment planning CT images can improve the CBCT image quality and can guarantee high positional accuracy, which is expected to be especially valuable for high-precision radiotherapy.

One method of improving image-quality accuracy using the proposed method is to improve registration accuracy. Our proposed method improves image quality using only treatment planning CT images paired with target CBCT images. Because the training dataset is created from the target CBCT image and the paired treatment planning CT image and because SimpleNet is trained so that MSE between the two images in the training dataset is reduced, misalignment between image pairs adversely affects the SimpleNet training accuracy. Therefore, we expect to improve image quality by achieving higher alignment accuracy in locations where high image alignment accuracy cannot be guaranteed, such as cavities in the intestinal tract and soft tissues.

Several limitations of this study are noted. First, as in earlier studies [19, 44–47], we downsampled the treatment planning CT images to match the size of the treatment planning CT images to the CBCT images. Consequently, this approach prevented accurate evaluation of the correction effect on the treatment planning CT images. For that reason, this study was limited to similarity-based image evaluations: this study did not include assessments related to dose distribution. Future work should address this downsampling issue to enable dose distribution evaluations, which would lead to the advancement of research on one-shot learning. Second, the valuation was conducted using only CBCT images acquired on the day of the start of radiation therapy. During radiotherapy, X-rays are irradiated over several dozen days. The tumor size is expected to decrease as the number of X-ray irradiations increases. Consequently, it is expected that the acquired CBCT images will be anatomically more distant from the treatment planning CT images than the CBCT images acquired at the start of treatment. This distance can lead to lower registration accuracy, which can in turn decrease the performance of the proposed method. However, if FLASH treatment [48], which involves patient exposure to a high dose rate of X-rays only a few times, becomes a common form of radiation therapy, then the shortcomings of the proposed method can be expected to become less noticeable. In addition, Lemus et al. investigated air mapping errors on the dosimetric accuracy of prostate ART, reporting that, despite the large mapping errors observed, the effect of dosimetry on the accuracy of adaptive planning dose calculations for prostate treatment is unlikely to exceed a difference of 3% or more [49]. This report suggests that our proposed OSSR has great potential, especially for prostate treatment.

For this study, we developed a new one-shot image-quality improvement model based on ZSSR to improve CBCT image quality using only the target CBCT image and the paired treatment planning CT image. Our proposed method can improve image quality while preserving the CBCT image location information. This important feature makes it possible to correct the X-ray irradiation position in IGRT accurately. Similarly, by improving the CBCT image quality, where the images are also used in adaptive radiotherapy, it is possible to plan treatment as appropriate using high-quality images such as treatment planning CT images. Moreover, because our method requires no image other than the target CBCT image and the paired treatment planning CT image, our proposed method is applicable immediately to a new CBCT image as long as a paired treatment planning CT image is available.

Moreover, conventional methods such as CycleGAN are adversely affected by the domain shift problem, by which the model’s performance is degraded by the difference between training and evaluation data. That degradation hinders their clinical application [50, 51]. However, the proposed method, OSSR, can resolve this difficulty because it trains the model each time using only a pair of CBCT and treatment planning CT images. Our method can be anticipated for application to different areas such as the head, neck, and lungs if the accuracy of image registration is equal to or better than that achieved by this study. Our method, which can readily improve CBCT image quality using only one pair of CBCT-treatment planning CT images, is expected to improve radiotherapy accuracy.

## Conclusion

This study demonstrates that the proposed “one-shot” super-resolution method based on ZSSR can improve CBCT image quality considerably using only the target CBCT image and its paired treatment planning CT image. This approach requires minimal training data, in contrast to conventional deep learning methods. The OSSR method achieved notable improvements in RMSE, PNSR, SSIM, and NMI, matching the performance of CycleGAN but also providing greater data efficiency. These findings highlight the potential of OSSR for enhancing CBCT image quality using only limited training data.

## Supplementary Information

Below is the link to the electronic supplementary material.

Supplementary file1 (PDF 834 KB)

## Data Availability

The dataset used and analyzed for this study is available from the corresponding author upon reasonable request.
