# Supplementary material for: Cone Beam Computed Tomography Image-Quality Improvement Using “One-Shot” Super-resolution
Source: J Imaging Inform Med. 2024 Dec 4;38(4):2120–33. doi: 10.1007/s10278-024-01346-w (PMC12344046; doi:10.1007/s10278-024-01346-w)
Supplement: Supplementary file 1 — Supplementary file1 (PDF 834 KB) [file 10278_2024_1346_MOESM1_ESM.pdf]

Supplementary Information

Network structure and loss functions of CycleGAN

Following the approach presented by Liang et al. [1], who replaced the two domains of the original CycleGAN [2] with CBCT and treatment planning CT images, we applied CycleGAN using this formalism.

The CycleGAN network structure comprises two generators and two discriminators. The generator is a Unet [3] based structure, which means that the output image size is equal to the input image size when converted to another domain. The discriminator is a PatchGAN [4] structure. Supplementary Tables 1, 2, and 3 present details of the CycleGAN network structure.

We designate the generator which generates treatment planning CT images from CBCT images as G_{PCT} and the generator which generates CBCT images from treatment planning CT images as G_{CBCT}. Discriminator D_{PCT} identifies treatment planning CT images as label 1 and treatment planning CT images generated from G_{PCT} as label 0. Discriminator D_{CBCT} identifies CBCT images as label 1 and CBCT images generated from G_{CBCT} as label 0.

The loss function used for this analysis is set up by replacing the original CycleGAN with the CBCT and treatment planning CT images proposed by Liang et al. [1]. This loss function comprises three parts: adversarial loss, cycle consistency loss, and identity mapping loss.

Adversarial loss is the loss of mapping the distribution of the generator image to the distribution of the target image and conversing with both the generator and the discriminator. Setting the set of CBCT images as $\mathbf{CBCT}$ and the set of treatment planning CT images as $\mathbf{CBCT}$, then G_{PCT} and D_{PCT} are aimed respectively at minimizing $\mathcal{L}_{\mathrm{GAN}-G_{\mathrm{PCT}}}$ and $\mathcal{L}_{\mathrm{GAN}-D_{\mathrm{PCT}}}$. These loss functions are formulated as

$$\mathcal{L}_{\mathrm{GAN}-G_{\mathrm{PCT}}} = \sum_{x \in \mathbf{CBCT}} \left(1 - D_{\mathrm{PCT}}\left(G_{\mathrm{PCT}}(x)\right)\right)^2, \quad (1)$$

$$\mathcal{L}_{\mathrm{GAN}-D_{\mathrm{PCT}}} = \sum_{y \in \mathbf{PCT}} \left(1 - D_{\mathrm{PCT}}(y)\right)^2 + \sum_{x \in \mathbf{CBCT}} D_{\mathrm{PCT}}\left(G_{\mathrm{PCT}}(x)\right)^2. \quad (2)$$

Similarly, G_{CBCT} and D_{CBCT} are aimed respectively at minimizing $\mathcal{L}_{\mathrm{GAN}-G_{\mathrm{CBCT}}}$ and $\mathcal{L}_{\mathrm{GAN}-D_{\mathrm{CBCT}}}$. These loss functions are formulated as

$$\mathcal{L}_{\mathrm{GAN}-G_{\mathrm{CBCT}}} = \sum_{y \in \mathbf{PCT}} \left(1 - D_{\mathrm{CBCT}}\left(G_{\mathrm{CBCT}}(y)\right)\right)^2, \tag{3}$$

and

$$\mathcal{L}_{\mathrm{GAN}-D_{\mathrm{CBCT}}} = \sum_{x \in \mathbf{CBCT}} \left(1 - D_{\mathrm{CBCT}}(x)\right)^2 + \sum_{y \in \mathbf{PCT}} D_{\mathrm{PCT}}\left(G_{\mathrm{PCT}}(y)\right)^2. \tag{4}$$

Cycle consistency loss is a loss by which G_{PCT} and G_{CBCT} conversion guarantee each other. To make the original input data and the recovered image approximately equal, the objective is to minimize

$$\mathcal{L}_{\mathrm{cycle-PCT}} = \sum_{y \in \mathbf{PCT}} \left|G_{\mathrm{PCT}}\left(G_{\mathrm{CBCT}}(y)\right) - y\right|, \tag{5}$$

and

$$\mathcal{L}_{\mathrm{cycle-CBCT}} = \sum_{x \in \mathbf{CBCT}} \left|G_{\mathrm{CBCT}}\left(G_{\mathrm{PCT}}(x)\right) - x\right|. \tag{6}$$

The identity mapping loss function preserves the pixel values between CBCT images and generated CBCT images, and those between treatment planning CT images and the generated treatment planning CT images. Consequently, the identity mapping losses for treatment planning CT and CBCT images are

$$\mathcal{L}_{\mathrm{identity-PCT}} = \sum_{y \in \mathbf{PCT}} |G_{\mathrm{PCT}}(y) - y|, \tag{7}$$

and

$$\mathcal{L}_{\mathrm{identity-CBCT}} = \sum_{x \in \mathbf{PCT}} |G_{\mathrm{CBCT}}(x) - x|. \tag{8}$$

Combining all these loss functions presented above, the loss functions of the generator are

$$\mathcal{L}_G = \mathcal{L}_{\mathrm{GAN}-G_{\mathrm{PCT}}} + \mathcal{L}_{\mathrm{GAN}-G_{\mathrm{CBCT}}} + \alpha\left(\mathcal{L}_{\mathrm{cycle-PCT}} + \mathcal{L}_{\mathrm{cycle-CBCT}}\right) + \beta\left(\mathcal{L}_{\mathrm{identity-PCT}} + \mathcal{L}_{\mathrm{identity-CBCT}}\right), \tag{9}$$

where $\alpha = 10$ and $\beta = 5$ were set as described by Liang et al. [1]. The loss function of the discriminator is

$$\mathcal{L}_D = \mathcal{L}_{\mathrm{GAN}-D_{\mathrm{PCT}}} + \mathcal{L}_{\mathrm{GAN}-D_{\mathrm{CBCT}}}. \tag{10}$$

Supplementary Table 1 Detailed network structure of generator in CycleGAN

Layer	Kernel size	Stride	Padding	Output size (*ch*, *h*, *w*)
Input	-	-	-	(1, 384, 384)
ReflectionPad	-	-	(3, 3)	(1, 390, 390)
Conv1+InstanceNorm+ReLU	(4, 4)	(2, 2)	(1, 1)	(64, 384, 384)
Conv2+InstanceNorm+ReLU	(4, 4)	(2, 2)	(1, 1)	(128, 192, 192)
Conv3+InstanceNorm+ReLU	(4, 4)	(2, 2)	(1, 1)	(256, 96, 96)
ResidualBlock1	-	-	-	(256, 96, 96)
ResidualBlock2	-	-	-	(256, 96, 96)
ResidualBlock3	-	-	-	(256, 96, 96)
ResidualBlock4	-	-	-	(256, 96, 96)
ResidualBlock5	-	-	-	(256, 96, 96)
ResidualBlock6	-	-	-	(256, 96, 96)
ResidualBlock7	-	-	-	(256, 96, 96)
ResidualBlock8	-	-	-	(256, 96, 96)
ResidualBlock9	-	-	-	(256, 96, 96)
TransposedConv1+InstanceNorm+ReLU	(3, 3)	(2, 2)	(1, 1)	(128, 192, 192)
TransposedConv2+InstanceNorm+ReLU	(3, 3)	(2, 2)	(1, 1)	(64, 384, 384)
ReflectionPad	-	-	(3, 3)	(64, 390, 390)
Conv4+Tanh	(7, 7)	(1, 1)	(1, 1)	(1, 384, 384)
Output	-	-	-	(1, 384, 384)

Supplementary Table 2 Detailed network structure of ResidualBlock in Generator

Layer	Kernel size	Stride	Padding	Output size (*ch*, *h*, *w*)
Input	-	-	-	(256, *h*, *w*)
ReflectionPad	-	-	(1, 1)	(256, *h*, *w*)
Conv1+InstanceNorm+ReLU	(3, 3)	(1, 1)	(1, 1)	(256, *h*, *w*)
ReflectionPad	-	-	(1, 1)	(256, *h*, *w*)
Conv2+InstanceNorm	(3, 3)	(1, 1)	(1, 1)	(256, *h*, *w*)
Residual	-	-	-	(256, *h*, *w*)
Output	-	-	-	(256, *h*, *w*)

Supplementary Table 3 Detailed network structure of the Discriminator in CycleGAN

Layer	Kernel size	Stride	Padding	Output size (*ch*, *h*, *w*)
Input	-	-	-	(1, 384, 384)
Conv1+LeakyReLU	(4, 4)	(2, 2)	(1, 1)	(64, 192, 192)
Conv2+InstanceNorm+LeakyReLU	(4, 4)	(2, 2)	(1, 1)	(128, 96, 96)
Conv3+InstanceNorm+LeakyReLU	(4, 4)	(2, 2)	(1, 1)	(256, 48, 48)
Conv4+InstanceNorm+LeakyReLU	(4, 4)	(1, 1)	(1, 1)	(512, 47, 47)
Conv5	(4, 4)	(1, 1)	(1, 1)	(1, 46, 46)
Average pool	(46, 46)	(1, 1)	-	(1, 1)
Output	-	-	-	(1, 1)

Quantitative evaluation for each patient

Supplementary Table 4 Quantitative evaluation of the positioning accuracy

NMI				
Patient ID	PlanCT	TVD	CycleGAN	OSSR
Pt01	0.426 (0.419–0.438)	0.507 (0.501–0.515)	0.486 (0.474–0.497)	0.569 (0.521–0.756)
Pt02	0.447 (0.419–0.455)	0.503 (0.492–0.511)	0.503 (0.493–0.521)	0.598 (0.527–0.850)
Pt03	0.460 (0.440–0.470)	0.514 (0.492–0.527)	0.575 (0.550–0.590)	0.574 (0.534–0.612)
Pt04	0.436 (0.406–0.451)	0.499 (0.492–0.506)	0.502 (0.467–0.545)	0.547 (0.473–0.689)
Pt05	0.435 (0.403–0.450)	0.501 (0.483–0.509)	0.522 (0.512–0.532)	0.617 (0.519–0.914)
Pt06	0.431 (0.404–0.442)	0.499 (0.492–0.514)	0.492 (0.484–0.504)	0.555 (0.512–0.646)
Pt07	0.448 (0.420–0.458)	0.497 (0.490–0.507)	0.511 (0.493–0.572)	0.558 (0.503–0.803)
Pt08	0.446 (0.424–0.455)	0.513 (0.502–0.518)	0.555 (0.526–0.574)	0.564 (0.517–0.757)
Pt09	0.434 (0.398–0.450)	0.501 (0.483–0.509)	0.502 (0.497–0.509)	0.607 (0.496–0.812)
Pt10	0.414 (0.382–0.423)	0.492 (0.483–0.496)	0.472 (0.466–0.484)	0.553 (0.485–0.679)
Pt11	0.429 (0.420–0.436)	0.504 (0.488–0.518)	0.539 (0.526–0.562)	0.558 (0.495–0.890)
Pt12	0.456 (0.439–0.470)	0.516 (0.509–0.524)	0.487 (0.465–0.497)	0.569 (0.525–0.643)
Pt13	0.456 (0.434–0.467)	0.512 (0.507–0.520)	0.507 (0.497–0.519)	0.629 (0.534–0.814)
Pt14	0.394 (0.347–0.408)	0.472 (0.461–0.483)	0.510 (0.498–0.532)	0.552 (0.454–0.774)
Pt15	0.452 (0.428–0.464)	0.508 (0.493–0.520)	0.489 (0.456–0.525)	0.609 (0.538–0.832)
Pt16	0.437 (0.412–0.451)	0.499 (0.486–0.515)	0.484 (0.474–0.494)	0.616 (0.491–0.884)

Pt17	0.432 (0.408–0.438)	0.500 (0.493–0.507)	0.472 (0.464–0.481)	0.578 (0.471–0.889)
Pt18	0.458 (0.434–0.472)	0.505 (0.481–0.516)	0.557 (0.533–0.595)	0.599 (0.542–0.804)
Pt19	0.406 (0.376–0.419)	0.472 (0.465–0.481)	0.493 (0.480–0.512)	0.526 (0.477–0.785)
Pt20	0.422 (0.397–0.437)	0.494 (0.478–0.503)	0.479 (0.472–0.495)	0.550 (0.497–0.730)
Pt21	0.459 (0.443–0.475)	0.519 (0.513–0.531)	0.546 (0.537–0.557)	0.566 (0.508–0.756)
Pt22	0.454 (0.436–0.462)	0.509 (0.501–0.519)	0.488 (0.473–0.504)	0.578 (0.526–0.749)
Pt23	0.470 (0.449–0.489)	0.514 (0.509–0.520)	0.543 (0.530–0.577)	0.599 (0.529–0.851)
Pt24	0.431 (0.408–0.448)	0.501 (0.483–0.508)	0.555 (0.531–0.571)	0.534 (0.462–0.671)
Pt25	0.458 (0.440–0.470)	0.514 (0.498–0.523)	0.497 (0.465–0.532)	0.578 (0.535–0.814)
Pt26	0.433 (0.398–0.451)	0.490 (0.465–0.506)	0.490 (0.475–0.499)	0.605 (0.485–0.941)
Pt27	0.418 (0.401–0.428)	0.496 (0.490–0.509)	0.461 (0.452–0.475)	0.545 (0.483–0.829)
Pt28	0.439 (0.407–0.456)	0.498 (0.489–0.511)	0.542 (0.508–0.564)	0.558 (0.499–0.695)
Pt29	0.471 (0.441–0.495)	0.523 (0.503–0.537)	0.555 (0.546–0.562)	0.637 (0.533–0.826)
Pt30	0.412 (0.386–0.422)	0.490 (0.474–0.501)	0.474 (0.464–0.483)	0.534 (0.445–0.777)

Values in the table show average values (min–max): PlanCT, treatment planning CT images after registration using B-spline deformable registration; CBCT images with total variation denoising; CycleGAN, synthetic PlanCT images converted from CBCT images by generating CycleGAN; OSSR, image-quality improvement images generated from CBCT images using the proposed method based on OSSR.

Supplementary Table 5 Quantitative evaluation of image quality improvement by RMSE

	RMSE			
Patient ID	CBCT	TVD	CycleGAN	OSSR
Pt01	13.32 (12.10–17.06)	12.57 (11.29–16.44)	12.49 (10.92–17.11)	11.70 (10.36–16.17)
Pt02	10.33 (9.13–13.00)	9.77 (8.49–12.40)	8.61 (6.98–11.64)	8.83 (7.22–11.87)
Pt03	11.85 (10.62–14.26)	11.13 (10.03–13.50)	10.84 (9.56–12.99)	10.13 (8.83–12.11)
Pt04	12.65 (10.57–17.02)	11.91 (10.07–16.08)	11.86 (9.56–16.60)	10.97 (9.24–14.88)
Pt05	11.50 (9.27–15.91)	10.80 (8.52–14.90)	10.22 (7.66–14.81)	9.64 (7.40–14.45)
Pt06	11.75 (10.63–17.91)	10.95 (9.91–17.00)	10.60 (9.35–17.06)	10.33 (9.11–15.41)
Pt07	10.31 (8.94–13.75)	9.59 (8.38–12.90)	9.48 (8.19–13.23)	8.97 (7.70–11.76)
Pt08	12.23 (10.53–16.33)	11.41 (9.82–15.23)	11.24 (9.29–15.96)	10.58 (8.79–14.79)
Pt09	11.51 (9.49–16.36)	10.79 (8.73–15.21)	10.42 (7.72–15.67)	9.49 (7.68–13.45)
Pt10	13.18 (11.59–20.92)	12.32 (10.79–20.04)	12.49 (10.47–20.19)	11.63 (9.89–18.40)
Pt11	13.72 (11.66–15.48)	12.85 (10.94–14.48)	12.72 (10.74–14.66)	11.82 (9.90–14.28)
Pt12	12.09 (10.78–14.97)	11.47 (10.23–14.25)	11.49 (10.04–13.71)	10.45 (9.21–13.06)
Pt13	10.34 (9.48–14.68)	9.93 (9.17–13.77)	7.98 (6.97–11.80)	8.65 (7.89–11.53)
Pt14	13.44 (11.09–22.67)	12.46 (9.96–21.74)	12.04 (9.65–22.02)	11.63 (9.17–21.01)
Pt15	10.64 (9.31–12.58)	10.05 (8.80–11.96)	9.47 (7.93–12.04)	9.05 (7.89–11.61)
Pt16	11.31 (9.88–15.73)	10.76 (9.45–14.89)	9.69 (8.41–14.72)	9.84 (8.86–13.92)

Pt17	12.06 (10.70–15.40)	11.24 (9.84–14.30)	10.88 (9.27–14.26)	10.29 (9.02–12.63)
Pt18	10.03 (9.09–11.89)	9.50 (8.57–11.39)	8.58 (7.06–10.86)	8.51 (7.12–10.91)
Pt19	12.03 (10.50–16.58)	11.02 (9.48–15.20)	11.09 (9.45–16.31)	10.09 (8.54–14.20)
Pt20	12.15 (11.26–16.14)	11.42 (10.44–15.20)	11.31 (10.06–15.60)	10.49 (9.07–14.28)
Pt21	11.75 (9.66–15.09)	10.94 (8.86–14.23)	10.86 (8.70–13.90)	9.89 (8.05–12.80)
Pt22	10.81 (9.85–13.05)	10.10 (9.16–12.17)	9.79 (8.50–12.22)	8.99 (8.16–10.66)
Pt23	10.22 (8.15–12.87)	9.55 (7.78–11.93)	9.41 (7.29–12.08)	8.97 (6.91–11.32)
Pt24	12.28 (10.42–14.70)	11.27 (9.52–13.77)	11.59 (9.39–13.76)	10.74 (8.88–13.32)
Pt25	10.36 (8.88–13.05)	9.64 (8.31–12.10)	9.41 (7.84–12.19)	8.96 (7.82–11.04)
Pt26	10.72 (8.38–14.97)	9.95 (7.52–13.92)	9.79 (7.03–14.49)	9.29 (7.14–12.88)
Pt27	12.97 (11.65–17.27)	11.97 (10.65–16.06)	11.79 (10.32–16.07)	11.23 (9.92–14.32)
Pt28	10.54 (9.20–16.08)	9.89 (8.65–15.07)	9.36 (8.00–15.15)	9.23 (8.11–14.26)
Pt29	11.95 (8.08–15.71)	11.40 (7.78–15.07)	11.38 (7.94–15.27)	10.78 (7.15–15.05)
Pt30	12.97 (11.68–17.43)	12.03 (10.80–16.35)	12.43 (11.26–16.45)	11.26 (10.12–15.25)

Values in the table show average values (min–max): CBCT, low-resolution images (raw data); TVD, CBCT images with total variation denoising; CycleGAN, synthetic PlanCT images converted from CBCT images by generating CycleGAN; OSSR, image-quality improvement images generated from CBCT images using the proposed method based on OSSR.

Supplementary Table 6 Quantitative evaluation of image quality improvement by PSNR

	PSNR			
Patient ID	CBCT	TVD	CycleGAN	OSSR
Pt01	25.67 (23.49–26.47)	26.18 (23.81–27.07)	26.24 (23.47–27.37)	26.81 (23.96–27.82)
Pt02	27.89 (25.85–28.92)	28.37 (26.26–29.55)	29.51 (26.81–31.25)	29.27 (26.64–30.96)
Pt03	26.69 (25.05–27.61)	27.23 (25.52–28.11)	27.46 (25.86–28.52)	28.06 (26.47–29.21)
Pt04	26.15 (23.51–27.65)	26.68 (24.00–28.07)	26.74 (23.73–28.52)	27.40 (24.68–28.82)
Pt05	27.03 (24.10–28.79)	27.58 (24.67–29.53)	28.12 (24.72–30.45)	28.55 (24.94–30.75)
Pt06	26.76 (23.07–27.60)	27.37 (23.52–28.21)	27.66 (23.49–28.71)	27.87 (24.38–28.94)
Pt07	27.90 (25.37–29.10)	28.53 (25.92–29.66)	28.64 (25.70–29.87)	29.11 (26.72–30.41)
Pt08	26.44 (23.87–27.68)	27.04 (24.48–28.29)	27.20 (24.07–28.77)	27.72 (24.73–29.25)
Pt09	27.02 (23.85–28.59)	27.59 (24.49–29.31)	27.96 (24.23–30.38)	28.67 (25.56–30.42)
Pt10	25.79 (21.72–26.85)	26.38 (22.09–27.47)	26.26 (22.03–27.73)	26.88 (22.83–28.23)
Pt11	25.40 (24.34–26.80)	25.97 (24.92–27.35)	26.06 (24.81–27.51)	26.70 (25.03–28.22)
Pt12	26.50 (24.63–27.47)	26.96 (25.06–27.94)	26.95 (25.39–28.10)	27.77 (25.81–28.84)
Pt13	27.86 (24.80–28.60)	28.20 (25.35–28.89)	30.12 (26.69–31.26)	29.40 (26.89–30.19)
Pt14	25.64 (21.02–27.23)	26.31 (21.39–28.17)	26.62 (21.28–28.44)	26.93 (21.68–28.88)
Pt15	27.61 (26.13–28.75)	28.11 (26.58–29.24)	28.63 (26.52–30.14)	29.02 (26.84–30.19)
Pt16	27.09 (24.20–28.24)	27.52 (24.67–28.62)	28.46 (24.77–29.63)	28.30 (25.26–29.18)

Pt17	26.53<br>(24.38–27.55)	27.13<br>(25.02–28.27)	27.44<br>(25.05–28.79)	27.92<br>(26.10–29.03)
Pt18	28.12<br>(26.63–28.96)	28.59<br>(27.00–29.47)	29.52<br>(27.42–31.16)	29.57<br>(27.38–31.09)
Pt19	26.58<br>(23.74–27.70)	27.35<br>(24.49–28.59)	27.32<br>(23.88–28.62)	28.11<br>(25.08–29.50)
Pt20	26.46<br>(23.97–27.10)	27.00<br>(24.50–27.76)	27.10<br>(24.27–28.08)	27.75<br>(25.04–28.98)
Pt21	26.80<br>(24.56–28.43)	27.42<br>(25.07–29.18)	27.49<br>(25.27–29.34)	28.31<br>(25.99–30.01)
Pt22	27.48<br>(25.82–28.26)	28.06<br>(26.42–28.90)	28.34<br>(26.39–29.55)	29.08<br>(27.58–29.90)
Pt23	27.99<br>(25.94–29.91)	28.57<br>(26.60–30.31)	28.71<br>(26.49–30.88)	29.11<br>(27.05–31.35)
Pt24	26.37<br>(24.78–27.77)	27.12<br>(25.35–28.56)	26.88<br>(25.36–28.68)	27.54<br>(25.64–29.16)
Pt25	27.87<br>(25.82–29.16)	28.50<br>(26.48–29.74)	28.72<br>(26.41–30.24)	29.13<br>(27.27–30.26)
Pt26	27.66<br>(24.63–29.67)	28.32<br>(25.26–30.60)	28.52<br>(24.91–31.19)	28.90<br>(25.93–31.06)
Pt27	25.89<br>(23.38–26.81)	26.59<br>(24.02–27.59)	26.73<br>(24.01–27.86)	27.14<br>(25.01–28.20)
Pt28	27.72<br>(24.00–28.85)	28.27<br>(24.57–29.39)	28.77<br>(24.52–30.07)	28.88<br>(25.05–29.95)
Pt29	26.76<br>(24.21–29.98)	27.17<br>(24.57–30.31)	27.19<br>(24.45–30.14)	27.68<br>(24.58–31.04)
Pt30	25.91<br>(23.31–26.78)	26.56<br>(23.86–27.47)	26.27<br>(23.81–27.10)	27.13<br>(24.46–28.03)

Values in the table show average values (min–max): CBCT, low-resolution images (raw data); TVD, CBCT images with total variation denoising; CycleGAN, synthetic PlanCT images converted from CBCT images by generating CycleGAN; OSSR, image-quality improvement images generated from CBCT images using the proposed method based on OSSR.

Supplementary Table 7 Quantitative evaluation of image quality improvement by SSIM

SSIM				
Patient ID	CBCT	TVD	CycleGAN	OSSR
Pt01	0.894<br>(0.887–0.900)	0.912<br>(0.902–0.922)	0.907<br>(0.900–0.913)	0.918<br>(0.888–0.928)
Pt02	0.912<br>(0.892–0.923)	0.926<br>(0.910–0.937)	0.933<br>(0.914–0.943)	0.936<br>(0.902–0.951)
Pt03	0.921<br>(0.901–0.932)	0.929<br>(0.917–0.937)	0.934<br>(0.916–0.943)	0.936<br>(0.917–0.947)
Pt04	0.899<br>(0.881–0.908)	0.914<br>(0.903–0.924)	0.914<br>(0.890–0.927)	0.918<br>(0.884–0.932)
Pt05	0.901<br>(0.867–0.916)	0.919<br>(0.895–0.938)	0.925<br>(0.894–0.939)	0.924<br>(0.878–0.951)
Pt06	0.899<br>(0.879–0.906)	0.916<br>(0.901–0.927)	0.919<br>(0.898–0.926)	0.926<br>(0.885–0.934)
Pt07	0.910<br>(0.883–0.923)	0.925<br>(0.905–0.936)	0.929<br>(0.903–0.940)	0.935<br>(0.898–0.950)
Pt08	0.902<br>(0.880–0.912)	0.918<br>(0.901–0.931)	0.921<br>(0.899–0.931)	0.925<br>(0.897–0.940)
Pt09	0.900<br>(0.862–0.915)	0.919<br>(0.891–0.937)	0.922<br>(0.885–0.939)	0.924<br>(0.882–0.951)
Pt10	0.887<br>(0.873–0.897)	0.907<br>(0.897–0.918)	0.903<br>(0.889–0.914)	0.912<br>(0.861–0.928)
Pt11	0.895<br>(0.882–0.904)	0.913<br>(0.903–0.923)	0.911<br>(0.901–0.921)	0.918<br>(0.889–0.929)
Pt12	0.914<br>(0.896–0.926)	0.924<br>(0.911–0.935)	0.926<br>(0.908–0.940)	0.929<br>(0.908–0.944)
Pt13	0.923<br>(0.901–0.932)	0.930<br>(0.921–0.940)	0.942<br>(0.920–0.950)	0.941<br>(0.907–0.954)
Pt14	0.853<br>(0.808–0.871)	0.900<br>(0.859–0.921)	0.890<br>(0.848–0.905)	0.897<br>(0.803–0.927)
Pt15	0.916<br>(0.902–0.925)	0.926<br>(0.914–0.933)	0.930<br>(0.918–0.938)	0.936<br>(0.909–0.946)
Pt16	0.905<br>(0.885–0.915)	0.921<br>(0.910–0.929)	0.928<br>(0.908–0.938)	0.927<br>(0.901–0.943)

Pt17	0.891 (0.875–0.901)	0.916 (0.903–0.931)	0.916 (0.897–0.927)	0.920 (0.888–0.940)
Pt18	0.922 (0.904–0.932)	0.930 (0.917–0.943)	0.941 (0.925–0.951)	0.944 (0.920–0.955)
Pt19	0.870 (0.831–0.885)	0.903 (0.878–0.923)	0.902 (0.865–0.917)	0.912 (0.840–0.930)
Pt20	0.892 (0.871–0.907)	0.914 (0.898–0.933)	0.909 (0.890–0.923)	0.918 (0.890–0.938)
Pt21	0.917 (0.909–0.923)	0.930 (0.920–0.941)	0.931 (0.922–0.939)	0.933 (0.905–0.948)
Pt22	0.916 (0.902–0.924)	0.929 (0.918–0.938)	0.931 (0.917–0.939)	0.939 (0.920–0.948)
Pt23	0.927 (0.912–0.940)	0.933 (0.919–0.941)	0.940 (0.927–0.953)	0.944 (0.928–0.957)
Pt24	0.887 (0.869–0.906)	0.910 (0.891–0.930)	0.903 (0.885–0.923)	0.913 (0.883–0.935)
Pt25	0.921 (0.900–0.934)	0.929 (0.914–0.938)	0.932 (0.914–0.943)	0.941 (0.905–0.954)
Pt26	0.896 (0.860–0.917)	0.921 (0.896–0.941)	0.921 (0.888–0.942)	0.924 (0.875–0.952)
Pt27	0.879 (0.866–0.891)	0.910 (0.896–0.924)	0.905 (0.889–0.916)	0.912 (0.874–0.931)
Pt28	0.906 (0.877–0.912)	0.922 (0.909–0.930)	0.930 (0.901–0.937)	0.932 (0.884–0.941)
Pt29	0.928 (0.906–0.942)	0.932 (0.914–0.941)	0.938 (0.917–0.951)	0.936 (0.911–0.957)
Pt30	0.877 (0.842–0.891)	0.905 (0.884–0.916)	0.896 (0.863–0.911)	0.908 (0.878–0.921)

Values in the table show average values (min–max): CBCT, low-resolution images (raw data); TVD, CBCT images with total variation denoising; CycleGAN, synthetic PlanCT images converted from CBCT images by generating CycleGAN; OSSR, image-quality improvement images generated from CBCT images using the proposed method based on OSSR.

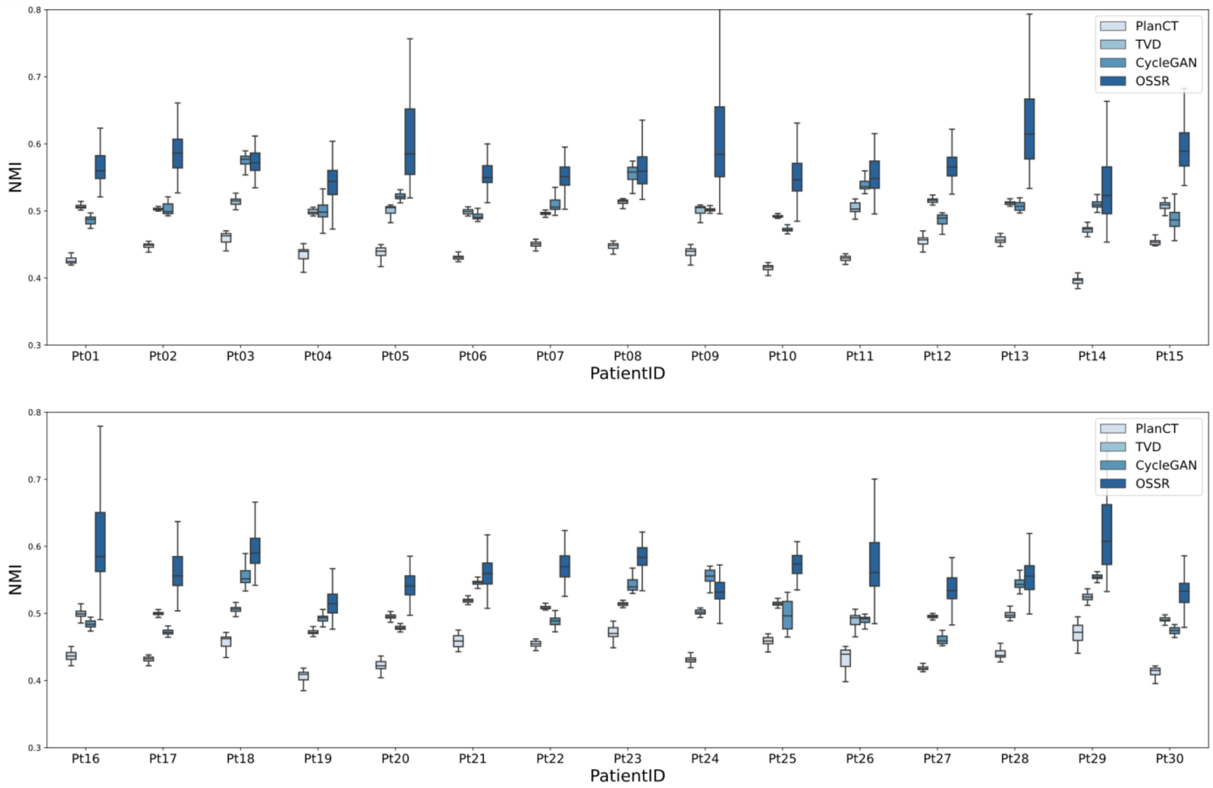

Supplementary Figure 1 Quantitative evaluation by NMI per patient. PlanCT label represents NMI between CBCT images before and treatment planning CT images after B-spline deformable registration. The TVD, CycleGAN, and OSSR labels respectively represent the NMI between the CBCT images and the images with improved quality achieved through the application of TVD, CycleGAN, and the proposed method.

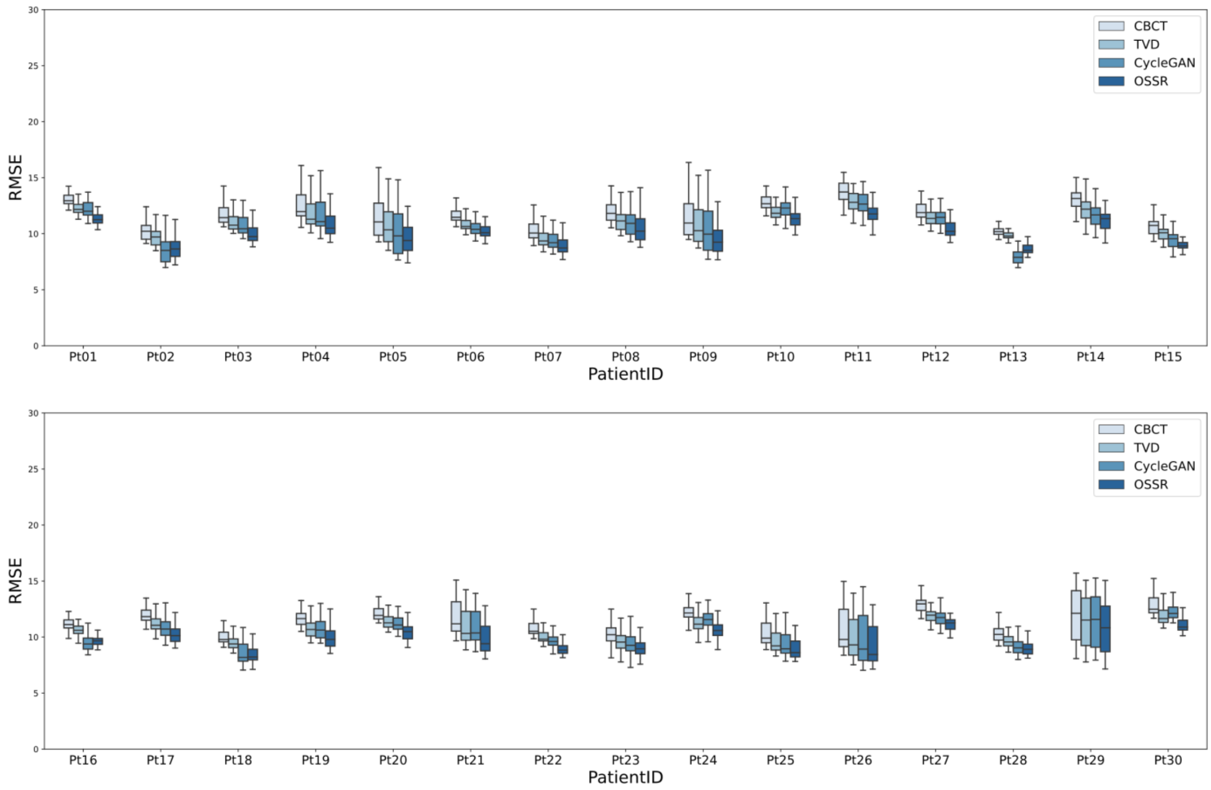

Supplementary Figure 2 Quantitative evaluation by RMSE per patient. The CBCT label represents RMSE between CBCT image and treatment planning CT images after B-spline deformable registration. The TVD, CycleGAN, and OSSR labels respectively represent the RMSE between the CBCT images and the images with improved quality achieved through the application of TVD, CycleGAN, and the proposed method.

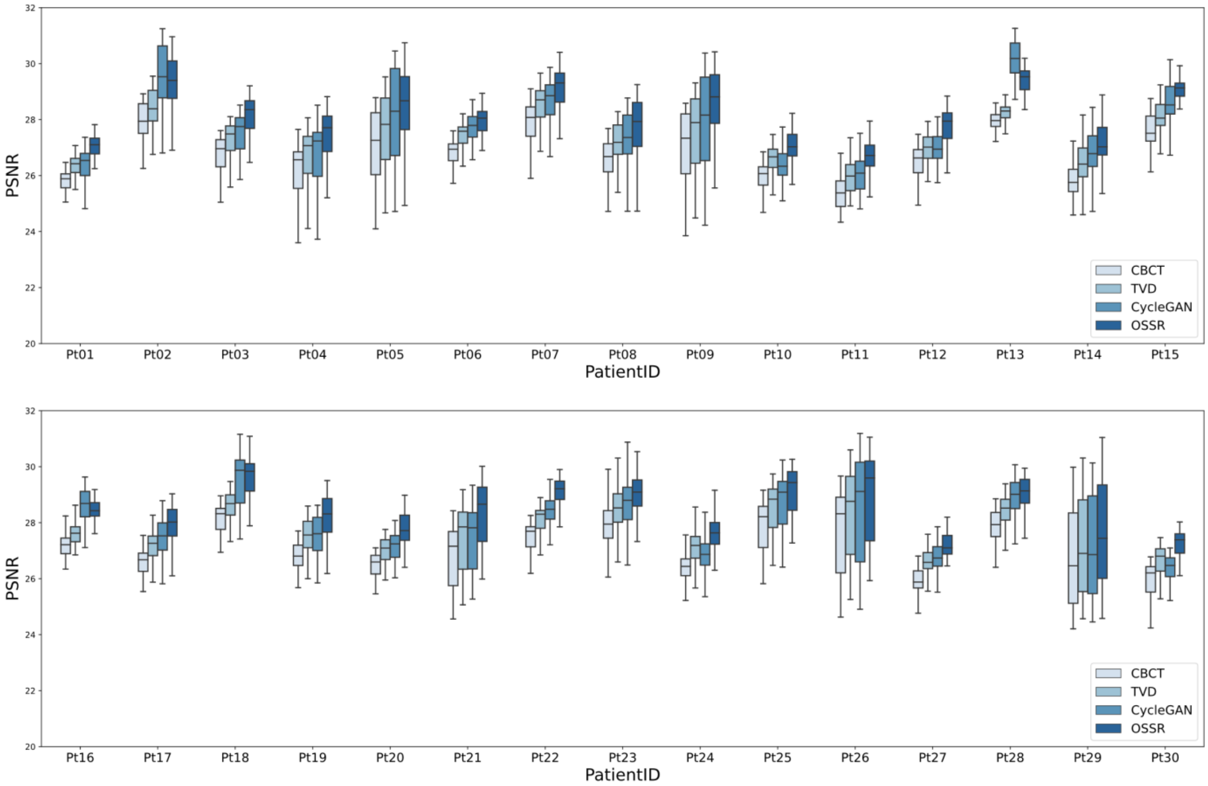

Supplementary Figure 3 Quantitative evaluation by PSNR per patient. The CBCT label represents PSNR between CBCT images and treatment planning CT images after B-spline deformable registration. The TVD, CycleGAN, and OSSR labels respectively denote the PSNR between the CBCT images and the images with improved quality achieved through the application of TVD, CycleGAN, and the proposed method.

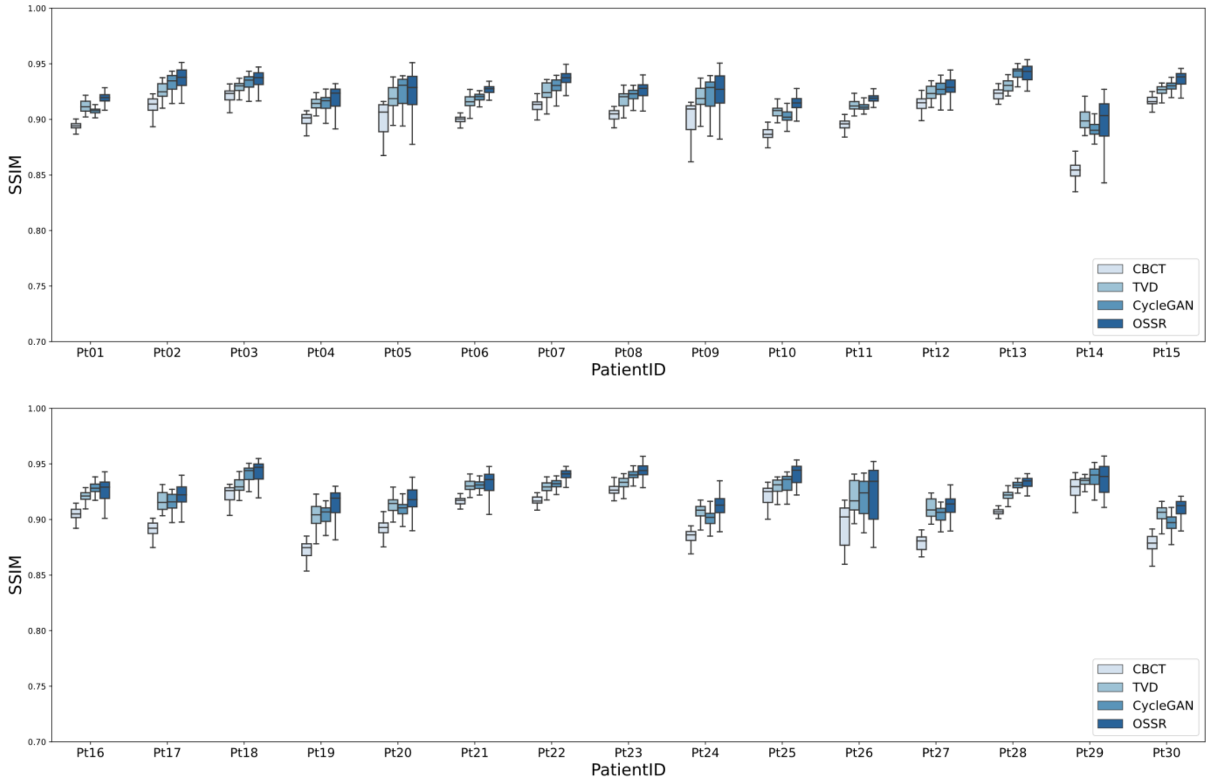

Supplementary Figure 4 Quantitative evaluation by SSIM per patient. The CBCT label represents SSIM between CBCT images and treatment planning CT images after B-spline deformable registration. The TVD, CycleGAn, and OSSR labels respectively denote the SSIM between the CBCT images and the images with improved quality achieved through the application of TVD, CycleGAN, and the proposed method.

References

1. Liang X, Chen L, Nguyen D, Zhou Z, Gu X, Yang M, et al.: Generating synthesized computed tomography (CT) from cone-beam computed tomography (CBCT) using CycleGAN for adaptive radiation therapy. Phys Med Biol 64:125002, 2019
2. Zhu JY, Park T, Isola P, Efros AA: Unpaired Image-to-Image Translation Using Cycle-Consistent Adversarial Networks. Proceedings of the IEEE International Conference on Computer Vision 2017: 2242-2251, 2017
3. Ronneberger O, Fischer P, Brox T: U-net: Convolutional networks for biomedical image segmentation. Lecture Notes in Computer Science (including subseries Lecture Notes in Artificial Intelligence and Lecture Notes in Bioinformatics) 9351:234-241, 2015
4. Isola P, Zhu J-Y, Zhou T, Efros AA, Research BA: Image-to-Image Translation with Conditional Adversarial Networks. In Proceedings of the IEEE Conference on Computer Vision and Pattern Recognition 2017:1125-1134, 2017
